# Supplementary material for: Adaptive strategies of high-flying migratory hoverflies in response to wind currents
Source: Proc Biol Sci. 2020 Jun 3;287(1928):20200406. doi: 10.1098/rspb.2020.0406 (PMC7341907; doi:10.1098/rspb.2020.0406)
Supplement: Supporting Figures and Tables [file rspb20200406supp1.docx]

**Figure S1.** Screenshots from a video of *Eristalis tenax* migration on the tip of the Karpaz Peninsula, north-east Cyprus on 2 May 2019, showing the consistently horizontal body axis of migrating hoverflies.


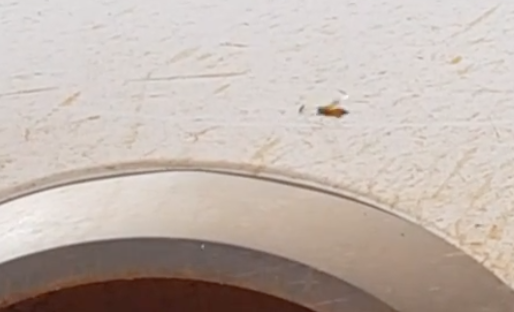

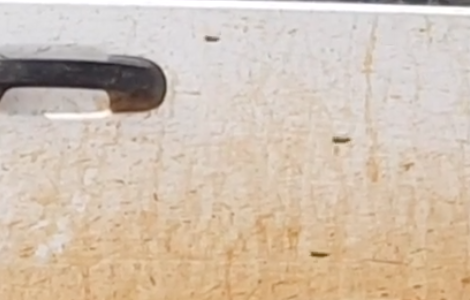

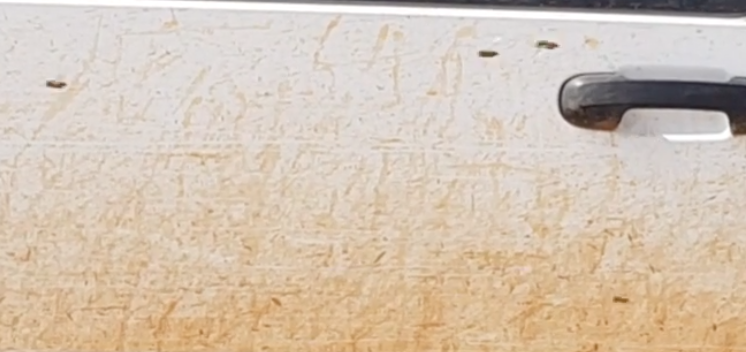

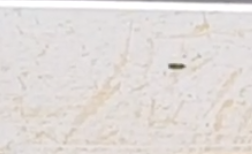


**Figure S2.** (*a – e*) Circular histograms of spring patterns of (*a*) the migration track directions of hoverfly mass migrations, (*b*) downwind directions during all days, (*c*) downwind directions during non-mass migration events, (*d*) downwind directions during mass migrations, and (*e*) flight headings during mass migrations exhibiting a significant degree of common orientation, for each of the three radar locations. Each small filled circle indicates the mean direction during a mass migration or a non-migration event. The bearing of the arrow indicates the mean direction of the entire dataset, while its length is proportional to the clustering of the dataset around the mean.


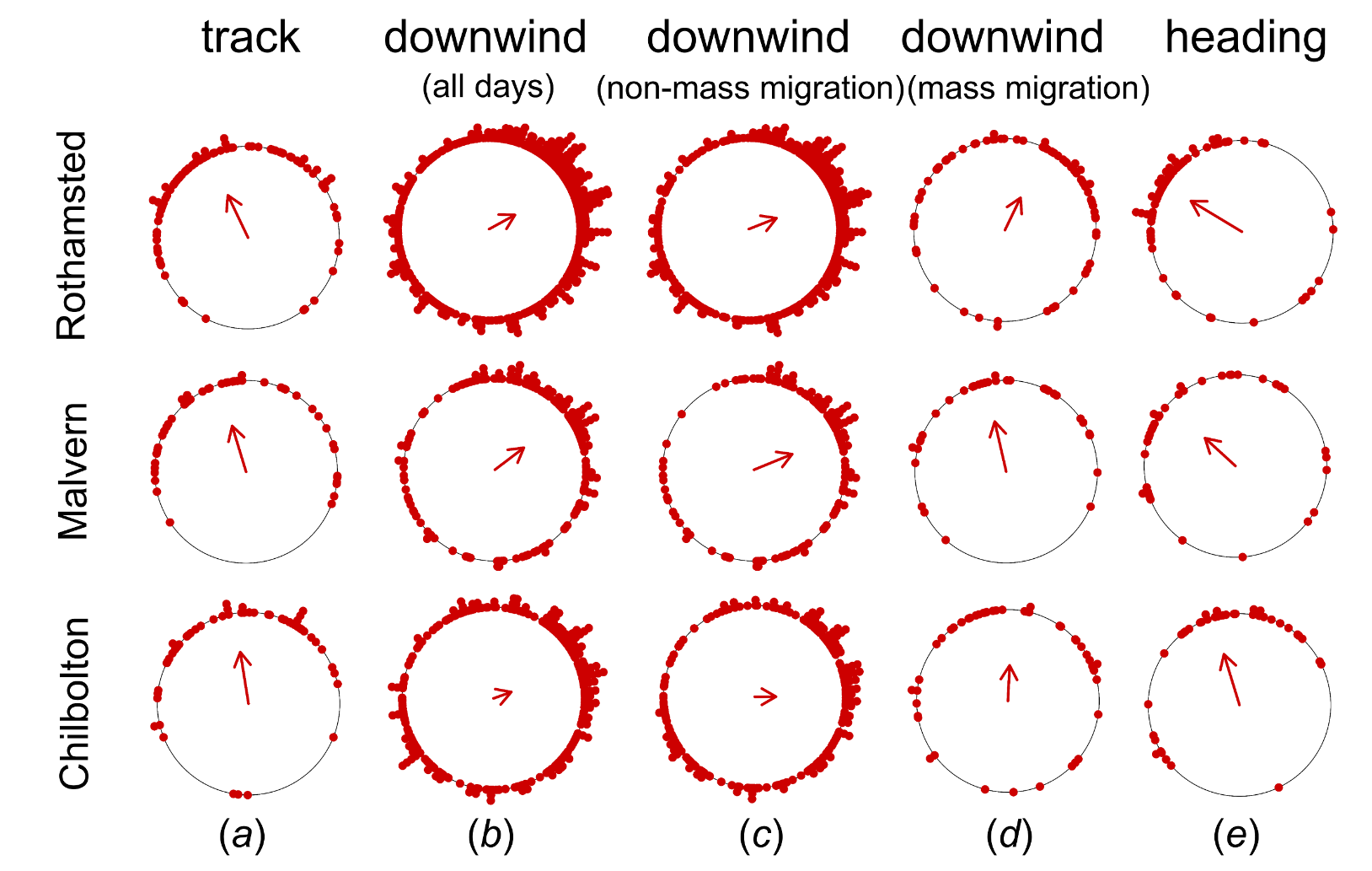


**Figure S3.** (*a – d*) Circular histograms of autumn patterns of (*a*) the migration track directions of hoverfly mass migrations, (*b*) downwind directions during all days, (*c*) downwind directions during non-mass migration events, (*d*) downwind directions during mass migrations, and (*e*) flight headings during mass migrations exhibiting a significant degree of common orientation, for each of the three radar locations. Each small filled circle indicates the mean direction during a mass migration or a non-migration event. The bearing of the arrow indicates the mean direction of the entire dataset, while its length is proportional to the clustering of the dataset around the mean; in cases where the distribution is not significantly different from random, no arrow is plotted.


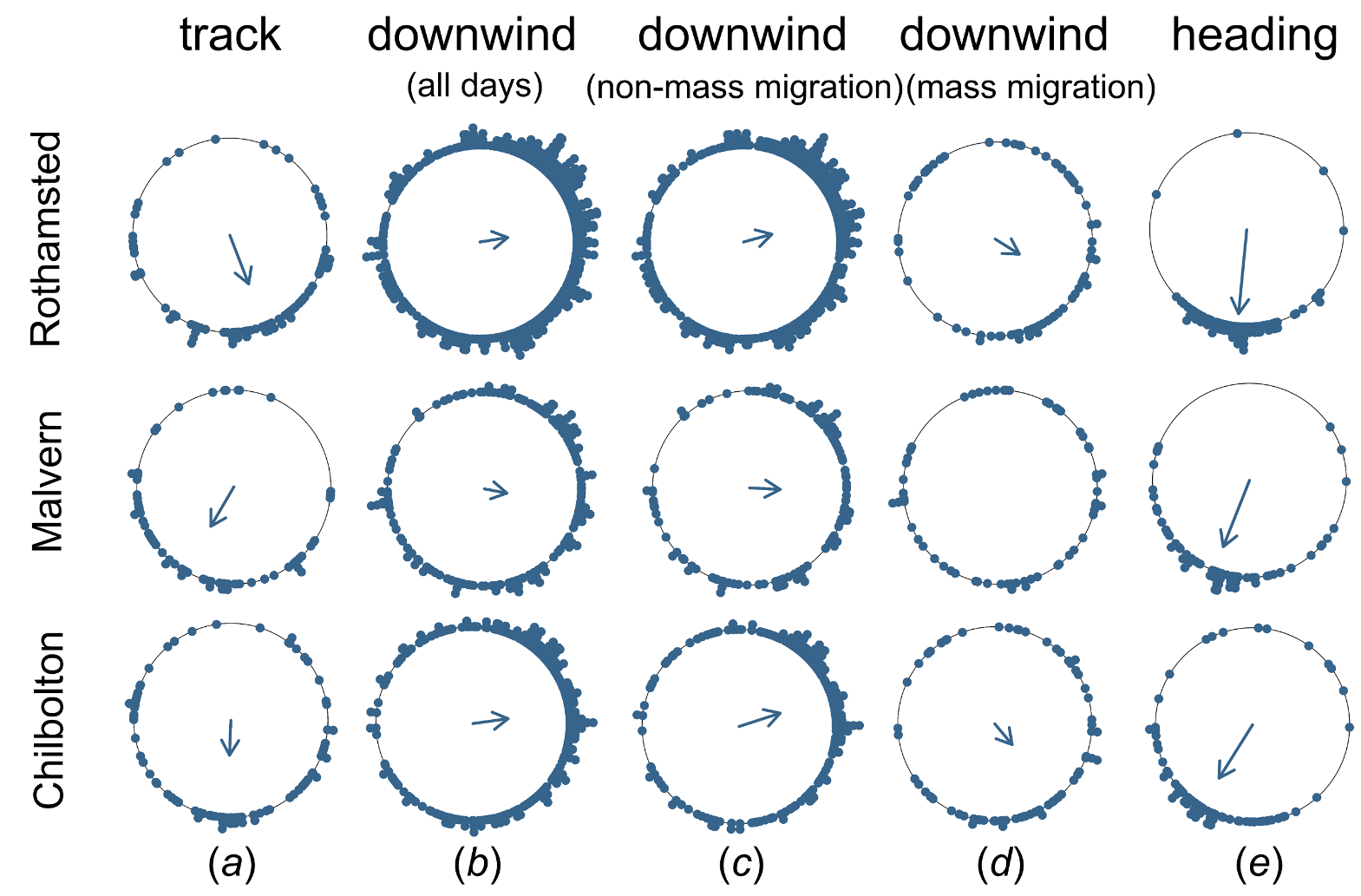


**Figure S4**. Diagrammatic representation of directional data during spring migrations of the hoverfly *Episyrphus balteatus*. The dashed grey line represents the presumed spring value of the PDM (preferred direction of movement), which we assume here is due north (0°). The blue arrow represented the self-powered heading vector, and the black arrow represents the track direction relative to the ground. The angle between the heading and the track is represented by *α*, which we call the ‘heading offset’; and the angle between the PDM and the track is represented by *Φ*, which we term the drift angle as it is a measure of how far the insect has been drifted off course by the wind. (a) In this case, the heading is on the anti-clockwise side of the track and so *α* would be assigned a negative value when used in the regression methodology of Green & Alerstam 2002 to find the slope and PDM (see Figure 2); but the heading is also closer to the PDM than the track, and so *α* would be assigned a positive value when used in the circular methodology to assess the degree of correction for wind drift (see Figure 3). (b) In this case, the heading is on the clockwise side of the track and so *α* would be assigned a positive value when used in the regression methodology of Green & Alerstam 2002 to find the slope and PDM (see Figure 2); but the heading is further away from the PDM than the track, and so *α* would be assigned a negative value when used in the circular methodology to assess the degree of correction for wind drift (see Figure 3).


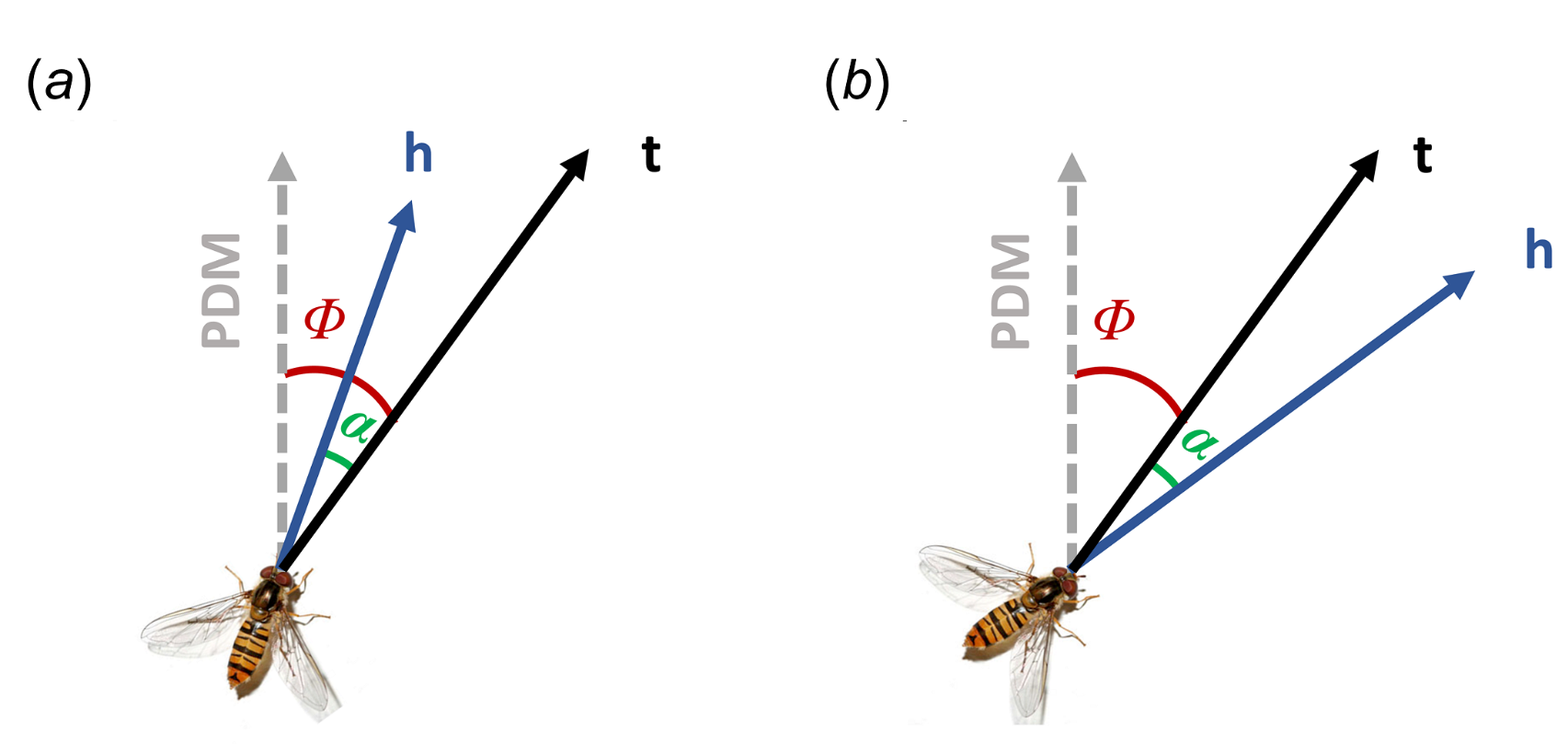


**Figure S5.** (*a – d*) Circular histograms of the migration track directions of all individual hoverflies in (*a*) spring (335°, n = 46,526, *r* = 0.45, *p* < 0.001) and (*c*) autumn (185°, n = 108,976, *r* = 0.38, *p* < 0.001); and flight headings of all individual hoverflies in (*b*) spring (322°, n = 40,018, *r* = 0.37, *p* < 0.001) and (*d*) autumn (198°, n = 107,291, *r* = 0.53, *p* < 0.001). Each coloured point indicates the direction of an individual hoverfly. The bearing of the arrow indicates the mean direction of the entire dataset, while its length is proportional to the clustering of the dataset around the mean.


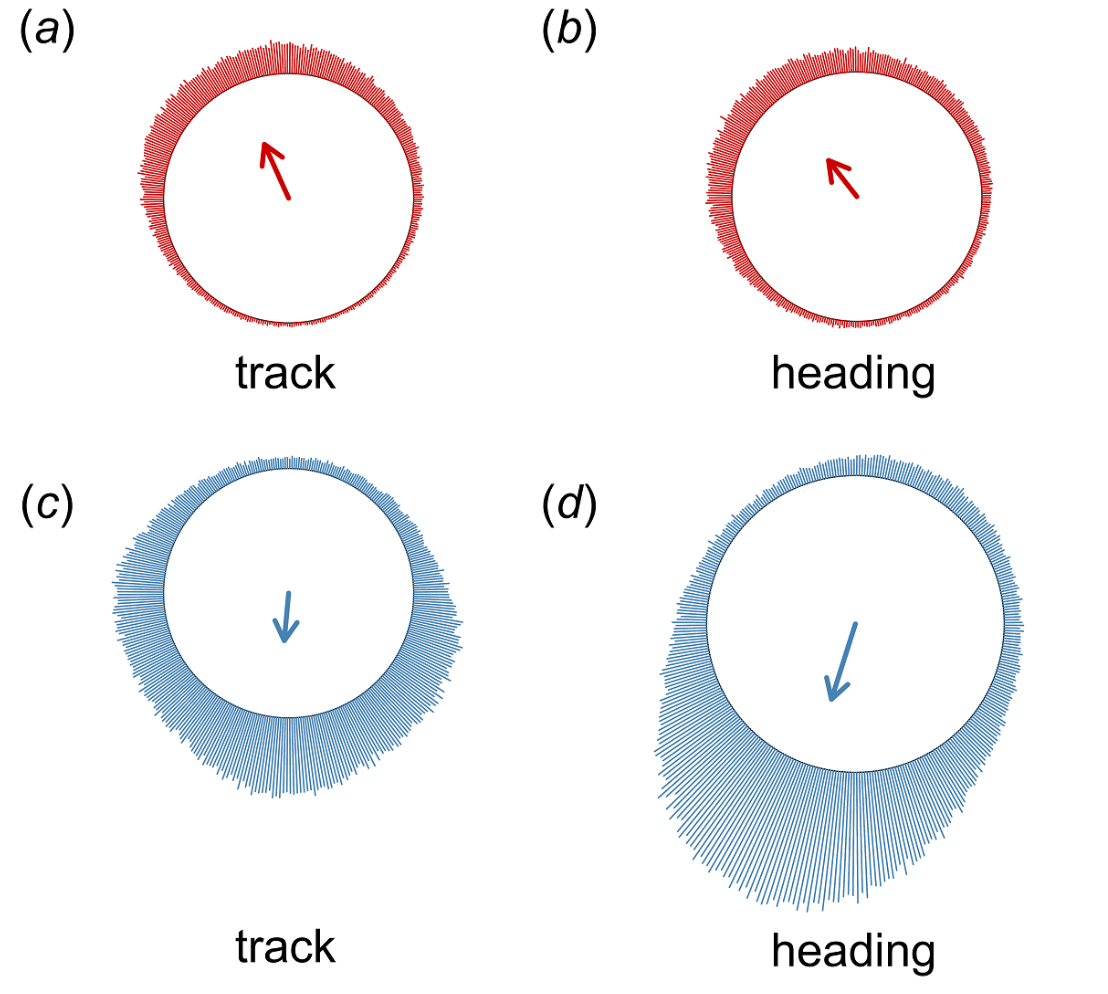


**Figure S6.**  Mean hourly track directions (a) and heading directions (b) of hoverfly mass migrations in the autumn. The dotted red horizontal lines show the overall mean value of (a) the autumn track (180°) and (b) the autumn heading (198°). The horizontal solid black lines represent the hourly mean and whiskers indicate the 5th and 95th percentiles.


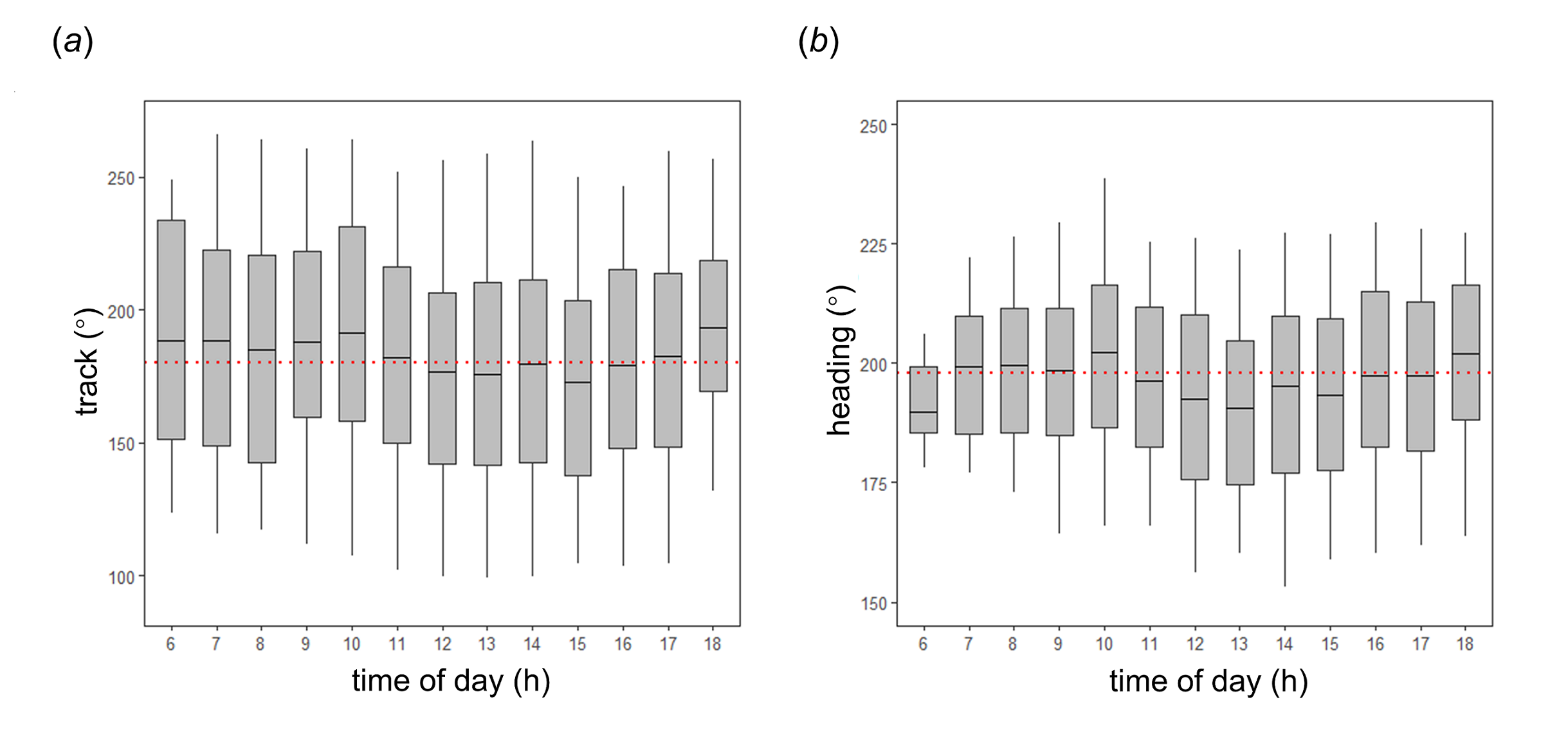


**Figure S7.** The distribution of wind speeds during all days in the study period with available data. The horizontal solid black line represents the median for each category, the dashed black line represents the mean, whiskers indicate the 5th and 95th percentiles, while the small circles show the outliers.


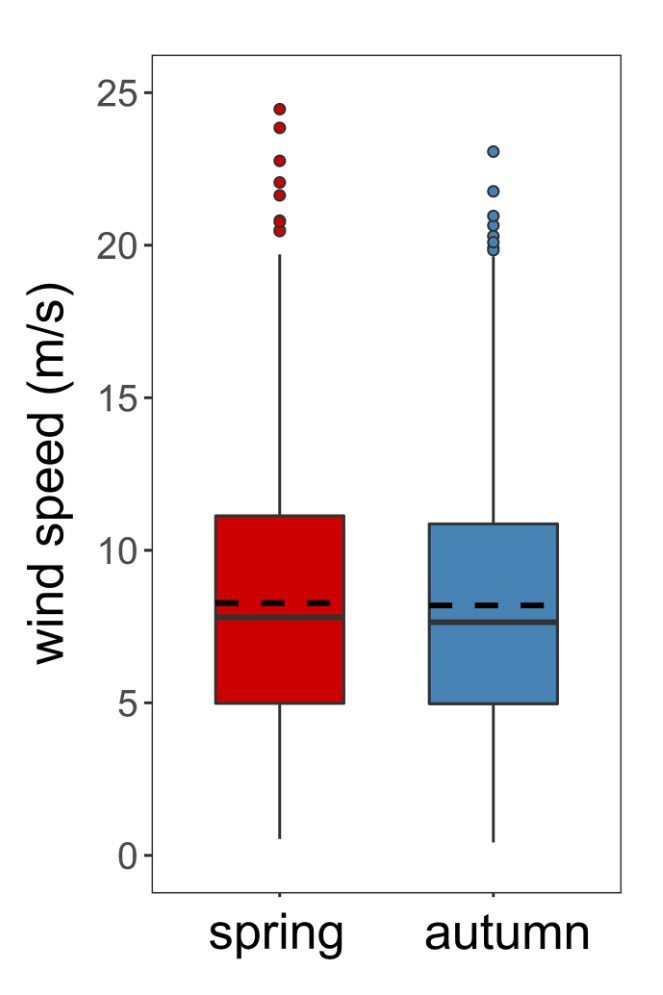


**Table S1.** Number of migration events and individual migrant hoverflies, and degree of common orientation, during spring and autumn migrations at each radar location.

| season | site | migration occasions | total hoverflies | mass migrations | total  hoverflies | common orientation | heading  r-value  (± s.d.) |
| --- | --- | --- | --- | --- | --- | --- | --- |
| spring | Rothamsted | 451 | 27,136 | 85 (19%) | 19,478  (72%) | 69/85  (81%) | 0.340± 0.126 |
|  | Chilbolton | 263 | 20,970 | 54 (21%) | 15,589  (74%) | 39/54  (72%) | 0.313 ± 0.126 |
|  | Malvern | 217 | 14,648 | 47 (22%) | 11,495  (78%) | 36/47  (77%) | 0.249 ± 0.100 |
|  | Total | 931 | 62,754 | 186 (20%) | 46,562 (75%) | 144/186 (77%) | 0.310 ± 0.125 |
| autumn | Rothamsted | 554 | 58,088 | 93 (17%) | 39,231  (68%) | 88/93  (95%) | 0.545 ± 0.134 |
|  | Chilbolton | 314 | 46,233 | 85 (27%) | 35,930  (78%) | 78/85  (92%) | 0.484 ± 0.166 |
|  | Malvern | 231 | 42,223 | 70 (30%) | 33,815  (80%) | 65/70  (93%) | 0.450 ± 0.162 |
|  | Total | 1,099 | 146,544 | 248 (23%) | 108,976 (75%) | 231/248 (93%) | 0.498 ± 0.158 |
| TOTAL |  | 2,030 | 209,298 | 434 (21%) | 155,538 (75%) | 375/434 (86%) |  |

**Table S2.** Mean track, heading and wind directions during spring and autumn migrations at each radar location. Non-significant distributions are highlighted in blue text.

| season | site | directional data | n | mean direction (°) | *r* | *p* |
| --- | --- | --- | --- | --- | --- | --- |
| spring | Rothamsted | track | 85 | 334.51 | 0.513 | <0.001 |
|  |  | wind (all days) | 465 | 61.32 | 0.322 | <0.001 |
|  |  | wind (non-mass migration) | 395 | 68.46 | 0.325 | <0.001 |
|  |  | wind (mass migration) | 70 | 25.71 | 0.392 | <0.001 |
|  |  | heading | 69 | 301.04 | 0.652 | <0.001 |
|  | Malvern | track | 47 | 343.08 | 0.524 | <0.001 |
|  |  | wind (all days) | 178 | 52.81 | 0.398 | <0.001 |
|  |  | wind (non-mass migration) | 145 | 67.77 | 0.451 | <0.001 |
|  |  | wind (mass migration) | 33 | 347.06 | 0.561 | <0.001 |
|  |  | heading | 36 | 312.42 | 0.452 | <0.001 |
|  | Chilbolton | track | 54 | 350.94 | 0.572 | <0.001 |
|  |  | wind (all days) | 264 | 70.24 | 0.206 | <0.001 |
|  |  | wind (non-mass migration) | 217 | 89.83 | 0.232 | <0.001 |
|  |  | wind (mass migration) | 47 | 2.75 | 0.389 | <0.001 |
|  |  | heading | 39 | 342.97 | 0.581 | <0.001 |
| autumn | Rothamsted | track | 93 | 158.85 | 0.537 | <0.001 |
|  |  | wind (all days) | 502 | 80.84 | 0.289 | <0.001 |
|  |  | wind (non-mass migration) | 429 | 74.62 | 0.302 | <0.001 |
|  |  | wind (mass migration) | 73 | 121.86 | 0.293 | 0.002 |
|  |  | heading | 88 | 185.58 | 0.869 | <0.001 |
|  | Malvern | track | 70 | 210.33 | 0.471 | <0.001 |
|  |  | wind (all days) | 190 | 100.74 | 0.236 | <0.001 |
|  |  | wind (non-mass migration) | 215 | 93.02 | 0.315 | <0.001 |
|  |  | wind (mass migration) | 61 | -- | -- | 0.440 |
|  |  | heading | 65 | 201.59 | 0.735 | <0.001 |
|  | Chilbolton | track | 85 | 182.14 | 0.359 | <0.001 |
|  |  | wind (all days) | 285 | 82.08 | 0.366 | <0.001 |
|  |  | wind (non-mass migration) | 129 | 72.05 | 0.443 | <0.001 |
|  |  | wind (mass migration) | 70 | 140.15 | 0.279 | 0.004 |
|  |  | heading | 78 | 212.10 | 0.648 | <0.001 |

**Table S3.** Watson-Wheeler tests between mean track, heading and wind directions during spring and autumn migrations for each radar location. Significant differences between pair-wise comparisons are highlighted in red text.

| season | group 1 | group 2 | directional data | *w* | *p* |
| --- | --- | --- | --- | --- | --- |
| spring | Rothamsted | Chilbolton | track | 4.015 | 0.134 |
|  |  |  | wind (all days) | 4.883 | 0.087 |
|  |  |  | wind (non-mass migration) | 4.520 | 0.104 |
|  |  |  | wind (mass migration) | 2.169 | 0.400 |
|  |  |  | heading | 26.265 | <0.001 |
|  | Rothamsted | Malvern | track | 0.171 | 0.918 |
|  |  |  | wind (all days) | 2.301 | 0.317 |
|  |  |  | wind (non-mass migration) | 2.911 | 0.233 |
|  |  |  | wind (mass migration) | 6.303 | 0.042 |
|  |  |  | heading | 4.686 | 0.096 |
|  | Chilbolton | Malvern | track | 2.235 | 0.327 |
|  |  |  | wind (all days) | 9.543 | 0.008 |
|  |  |  | wind (non-mass migration) | 8.686 | 0.013 |
|  |  |  | wind (mass migration) | 1.628 | 0.443 |
|  |  |  | heading | 8.223 | 0.016 |
| autumn | Rothamsted | Chilbolton | track | 6.844 | 0.033 |
|  |  |  | wind (all days) | 2.321 | 0.313 |
|  |  |  | wind (non-mass migration) | 5.530 | 0.063 |
|  |  |  | wind (mass migration) | 0.927 | 0.629 |
|  |  |  | heading | 32.358 | <0.001 |
|  | Rothamsted | Malvern | track | 15.651 | <0.001 |
|  |  |  | wind (all days) | 2.166 | 0.339 |
|  |  |  | wind (non-mass migration) | 1.343 | 0.511 |
|  |  |  | wind (mass migration) | -- | -- |
|  |  |  | heading | 11.967 | 0.002 |
|  | Chilbolton | Malvern | track | 6.176 | 0.046 |
|  |  |  | wind (all days) | 4.608 | 0.010 |
|  |  |  | wind (non-mass migration) | 4.008 | 0.135 |
|  |  |  | wind (mass migration) | -- | -- |
|  |  |  | heading | 4.230 | 0.121 |

**Table S4.** Mean track direction, mean heading and estimated PDM for each hour during hoverfly mass migrations.

|  | Mean track  (95% CI) | | Mean heading  (95% CI) | Number of events | Number of individuals |
| --- | --- | --- | --- | --- | --- |
| 6 a.m. - 7 a.m. | | 188.46  (123.36,249.19) | 189.97  (175.45,211.12) | 25 | 3161 |
| 7 a.m. - 8 a.m. | | 188.41  (115.94,265.92) | 199.30  (171.08,267.74) | 51 | 6028 |
| 8 a.m. - 9 a.m. | | 185.22  (117.13,264.00) | 199.51  (165.61,255.40) | 84 | 8147 |
| 9 a.m. - 10 a.m. | | 188.11  (111.68,260.90) | 198.38  (159.25,233.98) | 107 | 9357 |
| 10 a.m. - 11 a.m. | | 191.19  (107.61,263.96) | 202.27  (158.64,245.61) | 107 | 9670 |
| 11 a.m. - 12 a.m. | | 182.17  (102.16,252.10) | 196.36  (149.26,238.77) | 111 | 9858 |
| 12 a.m. - 1 p.m. | | 176.59  (99.74,256.39) | 192.37  (145.78,235.50) | 116 | 9857 |
| 1 p.m. - 2 p.m. | | 175.81  (93.39,258.61) | 190.60  (153.78,235.42) | 114 | 10676 |
| 2 p.m. - 3 p.m. | | 179.70  (99.73,263.87) | 195.15  (152.41,234.80) | 120 | 10611 |
| 3 p.m. - 4 p.m. | | 172.84  (104.55,250.18) | 193.28  (156.38,232.94) | 112 | 10131 |
| 4 p.m. - 5 p.m. | | 179.05  (103.39,246.44) | 197.40  (155.64,237.30) | 101 | 9266 |
| 5 p.m. - 6 p.m. | | 182.76  (104.33,259.55) | 197.28  (156.28,242.93) | 86 | 7423 |
| 6 p.m. -7 p.m. | | 193.45  (132.08,256.79) | 201.96  (163.80,233.41) | 56 | 4267 |

**Table S5.** Magnitude and direction of heading offsets during spring and autumn hoverfly mass migrations.

| season | drift angle  *Φ* (°) | n | ratio of positive to negative values of *α* | mean heading offset *α* (°) | 95% c.i. (°) | *p* |
| --- | --- | --- | --- | --- | --- | --- |
| spring | <10 | 15 | 11:4 (73%) | +13.84 | -8.23, +35.91 | >0.05 |
|  | >10 | 129 | 67:62 (52%) | +0.80 | -7.34, +8.95 | >0.05 |
|  | total | 144 | 73:71 (51%) | +2.16 | -5.44, +9.76 | >0.05 |
| autumn | <10 | 22 | 13:9 (59%) | +2.43 | -4.84, +9.71 | >0.05 |
|  | >10 | 209 | 182:27 (87%) | +30.42 | +25.96, +34.88 | <0.05 |
|  | total | 231 | 191:40 (83%) | +27.76 | +23.54, +31.98 | <0.05 |
